# Extracorporeal membrane oxygenation in near-drowning patients with cardiac or pulmonary failure

**DOI:** 10.1186/s13049-014-0077-8

**Published:** 2014-12-12

**Authors:** Kun Il Kim, Won Yong Lee, Hyoung Soo Kim, Jae Han Jeong, Ho Hyun Ko

**Affiliations:** Department of Cardiothoracic Surgery, Hallym University Dongtan Sacred Heart Hospital, Seoku-dong, Hwaseong-si, Gyeonggi-do 445-907 Korea; Department of Cardiothoracic Surgery, Hallym University Sacred Heart Hospital, 896, Pyeongchon-dong, Dongan-gu, Anyang-si, Gyeonggi-do 431-796 Korea; Department of Cardiothoracic Surgery, Hallym University Chuncheon Sacred Heart Hospital, Gyo-dong, Chuncheon-si, Gangwon-do 200-704 Korea

**Keywords:** Drowning, Near-drowning, Extracorporeal membrane oxygenation, Cardiac or pulmonary failure, Acute respiratory distress syndrome

## Abstract

**Background:**

The aim of this study was to determine the early outcomes of using extracorporeal membrane oxygenation (ECMO) in near-drowning patients with cardiac or pulmonary failure.

**Methods:**

This study was based on data from 9 patients including 2 children (mean age 33; 8 males, 1 female) who received ECMO after near-drowning between 2008 and 2013. Veno-arterial or veno-arteriovenous ECMO was used in 2 patients with sustained cardiac arrest and veno-venous ECMO was used in 7 patients with severe acute respiratory distress syndrome (ARDS). The means of the partial arterial oxygen pressure (PaO_2_), Murray score, sequential organ failure assessment (SOFA) score, and simplified acute physiology score II (SAPS-II) prior to ECMO were 59.7 ± 9.9 mmHg on 100% oxygen, 3.5 ± 0.6, 11.4 ± 1.9, and 73.0 ± 9.2, respectively.

**Results:**

The PaO_2_ mean improved to 182 ± 152 mmHg within 2 h post-ECMO. The mean of SOFA score and SAPS-II decreased significantly to 8.6 ± 3.2 (p = 0.013) and 46.4 ± 5.1 (p = 0.008), respectively, at 24 h post-ECMO with mean flow rate of 3.9 ± 0.8 l/min. ECMO was weaned at a mean duration of 188 (range, 43–672) h in all patients. Seven patients were discharged home without neurological sequelae, while 2 patients who had hypoxic brain damage died after further referral. The overall survival with favourable neurological outcomes at 3 months was 77.8%. There were no complications related to ECMO.

**Conclusions:**

ECMO was safe and effective for patients with ongoing cardiac arrest or ARDS after a near-drowning incident and can be used as a resuscitative strategy in near-drowning patients with cardiac or pulmonary failure resistant to conventional ventilator therapy.

## Background

According to the World Health Organization, more than 500,000 deaths each year and 0.7% of all deaths worldwide are due to unintentional drowning [1]. The outcome of patients after near-drowning is mainly related to anoxic encephalopathy. However, pulmonary oedema also commonly occurs after initial resuscitation, frequently progresses to acute respiratory distress syndrome (ARDS), and sometimes causes circulatory dysfunction and cardiac arrest. Conventional mechanical ventilation is usually used in patients with severe ARDS after drowning. However, this procedure may not effectively prevent hypoxemia or hypercapnia in these patients and may even exacerbate pulmonary damage due to barotrauma and a high concentration of inspired oxygen.

A previous study reported a survival rate of only 1.7% in 1,669 adult drowning patients who were in a cardiac arrest when the emergency medical services (EMS) arrived. Of these, only 0.7% survived with a favourable neurological outcome at 1-month after the near-drowning incident [2]. Despite the advances in critical and emergency care medicine, several attempts to resuscitation have proven unsuccessful in improving the outcomes of drowning and near-drowning victims [3,4].

Extracorporeal membrane oxygenation (ECMO) has been widely used for patients with cardiopulmonary failure, since the successful introduction for an adult with respiratory failure in 1972 and for neonates with respiratory failure in 1975 [5,6]. ECMO is a technique that provides lung rest by permitting reduced mechanical ventilator pressure and decreased concentration of inspired oxygen. It also allows cardiac rest while maintaining oxygenation, removal of carbon dioxide, and tissue perfusion. The development of a new resuscitative strategy such as ECMO is important to counter the exceedingly low survival rate of patients with cardiopulmonary failure after drowning. Nevertheless, there are very few case reports on the use of ECMO for resuscitation of drowning victims, especially adults.

Thus, the aim of this study was to present the early outcomes of ECMO used for near-drowning patients with cardiac or pulmonary failure.

## Methods

In this retrospective observational study, we reviewed the data of 9 patients including 2 children (mean age: 33 ± 17 years; 8 males, 1 female) who experienced near-drowning between 2008 and 2013. The ECMO program started in January 2006 at Hallym University Chuncheon Sacred Heart Hospital, which is located at the upper reaches of Han River in Korea, and included 172 patients for 8 years. This study was approved by the investigational review board of Hallym University Chuncheon Sacred Heart Hospital, and the need for informed consent was waivered.

### Patient characteristics at the scene and the emergency department

All the submersion incidents occurred in the fresh water during the summer, except one case wherein the subject fell into the freezing lake due to breakage of ice over the lake. The event was witnessed in all cases. The estimated submersion time was averaged at 6.0 ± 5.5 (range, 2–20) min. When rescued from the water, all subjects experienced loss of consciousness with no spontaneous breathing or palpable pulse. Three patients regained consciousness and exhibited recovery of spontaneous circulation (ROSC) promptly after initial resuscitation at the scene by laypersons. The other 6 patients underwent cardiopulmonary resuscitation (CPR) by the EMS. Thereafter, 4 of them achieved ROSC, and the other 2 sustained a cardiac arrest before arrival at the emergency department (ED). One of the 2 patients undergoing sustained CPR achieved ROSC at the ED. The patient characteristics and data related to drowning incident at the scene and ED are summarised in the Table 1.

**Table 1:** **Patient characteristics at the scene and the emergency department**

**Variable**	**1**	**2**	**3**	**4**	**5**	**6**	**7**	**8**	**9**
Age (years)	12	42	9	55	26	18	49	39	48
Sex	F	M	M	M	M	M	M	M	M
**Scene**									
Location of drowning	River	River	Pool	River	Lake	River	Pool	River	River
Precipitating events	no	Alcohol	no	Alcohol	no	no	Alcohol	Alcohol	Alcohol
Submersion duration (min)	2	7.5	3	5	20	5	5	2	5
Time interval to EMS arrival (min)	31	21	21	Unknown	31	5	21	22	57
CPR by EMS	n	y	y	y	y	n	y	y	n
**ED**									
Time interval to ED (min)	43	79	30	237	36	35	40	94	107
ROSC at arrival to ED	y	y	y	y	n	y	n	y	y
Level of consciousness at ED	Alert	Stupor	Stupor	Stupor	Coma	Alert	Coma	Alert	alert
GCS score	15	6	9	7	3	15	3	15	12
Pupil reflex	p	s	p	s	f	p	f	p	p
SaO_2_ (%)	88	58	91	76	Unchecked	78	72	68	75
Body temperature (°C)	36	36	35	35	Unchecked	37	34	36.8	34.5

F, female; M, male; min, minutes; EMS, emergency medical service; CPR, cardiopulmonary resuscitation; n, no; y, yes; ED, emergency department; ROSC, recovery of spontaneous circulation; GCS, Glasgow coma scale; p, prompt; s, sluggish; f, fixed; SaO_2_, arterial oxygen saturation.

### Management in the intensive care unit

On admission to the intensive care unit (ICU), 8 patients were placed on mechanical ventilator support because of hypoxemia. One patient with ongoing cardiac arrest received veno-arterial (VA) ECMO immediately. Within 6 h, 8 patients showed worsening of ARDS signs such as progressive hypoxemia, hypercapnia, and bilateral infiltrations on chest radiographs. These laboratory findings did not improve with conventional ventilator support. All patients were in a state of general anaesthesia with midazolam, fentanyl and vecuronium infusion if necessary, and controlled mandatory ventilation modes. The strategy of small tidal volume for ARDS was guarded in this study. Tidal volumes should not exceed 6 ml/kg with positive end expiratory pressure (PEEP) of 10–16 cmH_2_O. The plateau airway pressure should be maintained ≤ 30 cmH_2_O. The fraction of inspired oxygen (FiO_2_) was increased to 1.0 according to worsening hypoxemia. The volume controlled ventilation was applied initially and switched to the pressure controlled ventilation when the plateau airway pressure exceeded the established limit. This study cohort had experienced a near-drowning incident, loss of consciousness and cardiac arrest at the scene, and revealed unstable hemodynamic conditions and altered level of consciousness at the ER. The possibilities for the presence of the right ventricular dysfunction with pulmonary hypertension and the brain lesions with the increased intracranial pressure were not excluded. Therefore, the high PEEP level of 20–25 cmH_2_O, recruitment maneuver, permissive hypercapnia and prone position were not applied in the present study. Arterial blood gas analysis (ABGA) and relevant data are summarised according to time sequences in Table 2.

**Table 2:** **Patients’ laboratory findings and severity scores according to time sequences**

**Patients**	**1**	**2**	**3**	**4**	**5**	**6**	**7**	**8**	**9**	**Mean**	**SD**
Initial ABGA											
pH	7.28	7.24	6.93		6.8	7.3	6.99	7.23	7.23	7.13	0.19
PaCO_2_ (mmHg)	33	36	51		105	42	103	36	34	55.0	30.8
PaO_2_ (mmHg)	40	34	101		37	33	10	38	42	41.9	25.9
SaO_2_ (%)	67	53	92	48		56		60	66	63.1	14.4
ABGA after ventilator support											
pH	7.01	7.06	7.01	7.06		7.34		7.24	7.14	7.12	0.13
PaCO_2_	94	74	61	55		37		38	40	57.0	21.3
PaO_2_	67	57	50	39		155		69	52	69.9	38.9
PaO_2_/FiO_2_	67	57	50	39		155		69	52	69.9	38.9
ABGA prior to ECMO and severity scores											
pH	7.28	7.21	7.38	7.04	6.8	7.28	6.88	7.26	7.2	7.15	0.20
PaCO_2_	39	46	40	80	92	42	67	28	39	52.6	21.8
PaO_2_	69	65	39	58	71	64	75	59	64	62.7	10.4
PaO_2_/FiO_2_	69	65	39	58		64		59	64	59.7	9.9
Lactate (mmol/l)	6.3	8.1	10.2	6.7	15	5.9	9.8	5.5	5.2	8.1	3.2
Murray score	3.3	3.7	3.3	4	4	2.3	4	4	3	3.5	0.6
SOFA score	12	14	10	13	13	9	13	10	9	11.4	1.9
SAPS-II	58	81	76	81	76	67	84	73	61	73.0	9.2
ABGA after ECMO and severity scores											
pH	7.41	7.34	7.5	7.19	6.68	7.35	7.27	7.3	7.3	7.26	0.23
PaCO_2_	29	23	28	41	33	30	26	26	26	29.1	5.3
PaO_2_	87	92	97	82	548	250	243	138	102	182.1	152.1
PaO_2_/FiO_2_	290	153	149	182	1095	250	405	344	254	346.9	293.3
SaO_2_	97	99	98	94	100	100	100	99	98	98.3	1.9
lactate (24 h)	1.8	5.3		1.8	4.9	2.5	6	1.9	3.1	3.4	1.7
SOFA score (24 h)	8	15	9	11	10	5	5	7	7	8.6	3.2
SAPS-II (24 h)	45	55	47	45	51	38	48	41	48	46.4	5.1

SD, standard deviation; ABGA, arterial blood gas analysis; PaCO_2_, partial arterial carbon dioxide pressure; PaO_2_, partial arterial oxygen pressure; SaO_2_, arterial oxygen saturation; FiO_2_, the fraction of inspired oxygen; ECMO, extracorporeal membrane oxygenation SOFA, sequential organ failure assessment; SAPS-II, simplified acute physiology score; lactate, normal range 0.7-2.1 mmol/l; 24 h, 24 h post-ECMO.

ABGA prior to ECMO showed the following: pH, 7.15 ± 0.20; partial arterial carbon dioxide pressure (PaCO_2_), 52.6 ± 21.8 mmHg; and partial arterial oxygen pressure (PaO_2_) at FiO_2_ of 1.0, 59.7 ± 9.9 mmHg. Initially, the right lung fields were more severely affected in all patients except one. Thereafter, repeat chest radiographs showed rapidly progressing and widespread infiltrations in all 4 quadrants within 2 h except in one patient who had been transferred from a local hospital because of respiratory failure and stuporous level of consciousness (case 4 in Table 2). He showed severe hypercapnia and acidosis despite of conventional ventilator support. The mean Murray score prior to ECMO was 3.5 ± 0.6. The mean sequential organ failure assessment (SOFA) score and simplified acute physiology score II (SAPS-II) before ECMO were 11.4 ± 1.9 and 73.0 ± 9.2, respectively, with a predicted mortality rate of 87.1% by SAPS-II.

The inclusion criteria for ECMO in the present study were PaO_2_ < 70 mmHg for more than 2 h with FiO_2_ 1.0 and PEEP > 10 cmH_2_O, uncompensated respiratory acidosis with pH < 7.15, rapidly evolving diffuse infiltrates in all quadrant of chest radiographs, and unstable vital signs including cardiac arrest. ECMO was instituted within 2 h in 3 patients due to unstable vital signs and impending cardiac arrest.

### Statistical analysis

Statistical analysis was performed using SPSS version 21 (IBM Co., NY, USA). Continuous variables were analysed by Wilcoxon signed-rank sum test according to the variable characteristics and are expressed as mean with standard deviation. The level of statistical significance was set at P < 0.05.

## Results

The mean time between arrival at the ED and ECMO (door-to-ECMO time) was 187 ± 113 min. Femoral cannulation using Seldinger’s technique was attempted in all patients by using DLP and Bio-medicus cannulae with 17–24 Fr. for drainage and with 17–21 Fr. for return (Medtronic Inc., Minneapolis, MN, USA). Two types of centrifugal pumps and membrane oxygenators (MO) were used for the ECMO system: the Capiox Emergency Bypass System® (Terumo, Inc., Tokyo, Japan) with a polypropylene hollow-fibre MO, Capiox® RX25 (Terumo, Inc.) in the early period of this study and the PLS ECMO system® (Maquet Cardiopulmonary AG, Rastatt, Germany) with a solid polymethylpentene MO, Quadrox PLS (Maquet Cardiopulmonary AG) in the later period. The centrifugal pump speeds were adjusted to 2,000-3,500 rounds/min, providing a bypass blood flow rate of 50–80 ml/kg/min. Initially, the fresh gas flow through MO was started with an oxygen fraction of 1.0 and a flow rate of 6 l/min. The flow rate was then gradually adjusted to 4–8 l/min to allow normal removal of carbon dioxide. Ventilators were set to a tidal volume of 5 ml/kg, respiration rate (RR) of 10-12/min, PEEP of 4–8 cmH_2_O, and FiO_2_ of 0.21–0.6 for pulmonary rest. A haematocrit level > 33-35% and platelets count > 50,000–100,000/ml were achieved with transfusions when necessary. For anticoagulation, 3,000 units of heparin were infused just before cannulation. In 3 patients, the heparin doses were adjusted to maintain the activated clotting time at 160–180 s. In the other 6 patients, nafamostat mesilate (SK chemicals Life Science Biz., Seoul, Korea; licensed by Torii Pharmaceutical Co., Tokyo, Japan) was administered at 0.41-0.93 mg/kg/h according to the activated partial thromboplastin time with a target range of 60–80 s. Prophylactic antibiotics were used in all patients to avoid infectious complications.

The mean PaO_2_ increased to 182 ± 152 mmHg within 2 h with ECMO at a mean flow rate of 3.9 ± 0.8 l/min. Compared to the values prior to ECMO, the average SOFA score and SAPS-II at 24 h post-ECMO decreased significantly to 8.6 ± 3.2 (p = 0.013) and 46.4 ± 5.1 (p = 0.008), respectively; further, the predicted mortality rate calculated by SAPS-II decreased from 87.1% before ECMO to 37.0% with ECMO. VA ECMO was replaced by veno-venous (VV) ECMO after 60 h in one patient owing to improved circulatory function. Further, another patient who achieved ROSC at the ED received veno-arteriovenous (VAV) ECMO to prevent the recurrence of cardiac arrest, and then, VAV ECMO was converted to VV ECMO owing to the patient’s improved status. Moreover, one 18-year-old man underwent awake ECMO after 72 h. Since they had improved cardiopulmonary function and resolved pulmonary infiltrations, all patients were weaned from ECMO and mechanical ventilator at a mean duration of 188 (range, 42.5-672) and 122 (range, 58–429) h, respectively, except 1 patient who was given awake ECMO. ECMO was ceased when ABGA showed a pH of 7.35–7.45, PaO_2_ > 80 mmHg, and PaCO_2_ < 45 mmHg under the following conditions: gas blender FiO_2_ of 0.21, no sweep gas flow at an ECMO flow of 2 l/min, ventilator mode set to an FiO_2_ of 0.6, tidal volume of 6 ml/kg, PEEP of 8 cmH_2_O, and respiration rate (RR) of 12–16/min for VV ECMO (or 3 l/min of O_2_ via nasal prong for the patient who received awakening ECMO).

Two patients experienced hypoxic brain damage due to near-drowning. However, there were no complications related to ECMO. One child, who drowned in a swimming pool and developed prolonged pneumonitis, underwent a tracheostomy without an event. The average duration of ICU and hospital stays were 16 (range, 6–55) and 29 (range, 7–106) days. Seven patients with intact neurological outcomes were discharged home. Two patients with hypoxic brain damage were transferred to hospitals close to their residential areas, but eventually died. The overall mortality in this study at 3 months was 22.2%. The mean of the total hospital charges was 37,636 ± 26,193 (range, 9,923-98,627) US dollars (USD). The patients and their families paid 10,118 ± 8,840 USD, and the remainder of the hospital bill was charged to the National Health Insurance Service.

## Discussion

Drowning or near-drowning comprises a heterogeneous group of conditions with a wide spectrum of severity, and is difficult to standardise a definition and criteria for it. After drowning incidents, patients’ conditions range from asymptomatic to severe distress with multiorgan failure. Brain injury due to anoxia or hypoxia is a strong predictor of mortality after submersion, but pulmonary oedema is a commonly encountered after initial resuscitation. In some patients, a rapid improvement is seen but their condition may deteriorate suddenly within the first 12 h or up to several days later due to the development of pulmonary oedema and ARDS [7-10]. Therefore, severe pulmonary oedema and ARDS requiring mechanical ventilation is another predictor of mortality after drowning incidents. Szpilman revised the classification system of Menezes and Costa for drowning patients to stratify patients into 6 different risk subgroups, considering the initially obtained data on cardiopulmonary conditions, pulmonary auscultation with coughing, and arterial blood pressure. In his study including 1,831 drowning patients, the mortality rate was reported with a grade: grade 1 = 0%, grade 2 = 0.6%, grade 3 = 5.2%, grade 4 = 19.4%, grade 5 = 44%, and grade 6 = 93% [11]. A previous study reported 43 near-drowning cases, of which 48.8% required mechanical ventilation, and their mortality rate was 34.9% [12]. In another study of 1,737 patients who, at arrival of the EMS, were in a cardiac arrest due to drowning, the 1-month survival rate was 27.8, 9.4 and 1.7% in younger children (aged 0–4 years), older children (aged 5–17 years) and adults, respectively [2].

Severe pulmonary oedema can be partly explained as ischemia-reperfusion injury but may be aggravated by severe diastolic dysfunction and acute volume overload [3]. Often, pulmonary oedema progresses to ARDS, which causes circulatory dysfunction or cardiac arrest despite aggressive ventilator therapy. Considering the extremely low survival rate in drowning patients with severe pulmonary oedema and ARDS, a new resuscitative strategy such as ECMO needs to be developed to recover pulmonary and circulatory functions and improve the clinical outcomes.

ECMO is a technique that provides lung rest by permitting reduced ventilator settings and decreased concentration of inspired oxygen while maintaining oxygenation, removal of carbon dioxide, and tissue perfusion [13]. It also improves circulatory function and reduces the cardiac burden before the pulmonary function recovers. Currently, only few case reports are available on the use of ECMO in drowning patients, despite the brisk use and remarkable outcomes of ECMO in patients with ARDS. The Extracorporeal Life Support Organization (ELSO) has updated several guidelines for ECMO. The main indication for the use of ECMO in adult patients with respiratory failure is an 80% predicted mortality risk associated with a PaO_2_/FiO _2_ < 100 on FiO _2_ > 90% and/or a Murray score of 3–4 despite optimal care for > 6 h. Further, the indications include CO_2_ retention on mechanical ventilation despite high plateau pressure (>30 cmH_2_O) and immediate cardiac or respiratory collapse [14]. A previous retrospective review of the ELSO registry revealed 1,473 adults with respiratory failure, with an overall survival rate of 50% [15]. Lindskov and colleagues reported a 78% rate of weaning from ECMO and a 71% discharge rate in 124 patients with severe ARDS. They also evaluated the significance of the various severity-scoring systems and demonstrated that the SOFA score and SAPS-II were more strongly correlated with mortality than the Murray score and that a SOFA score > 20 and a SAPS-II > 70 were linked to mortality rates of 75% and 70%, respectively [16]. Severity-scoring systems such as the SAPS-II and SOFA have been commonly used to predict risks of mortality, compare ICU results, evaluate outcomes of a specific therapy, and stratify patients for clinical research. The SAPS-II can be calculated easily using programs on the internet and can estimate the predicted mortality.

On applying ABGA including the mean PaO_2_ of 59.7 mmHg on 100% oxygen and the mean Murray score of 3.5 in this study to the ELSO indications, our cohort fulfilled the ELSO criteria, although ECMO was initiated within 6 h owing to rapid deterioration of the patients’ status. When considering a mean SAPS-II of 73 prior to ECMO and a predicted mortality of 87%, a 100% rate of weaning from ECMO and discharge rate was remarkable, despite the small sample size. We also compared the SAPS-II and SOFA score before and after ECMO and found statistically significant decreases 24 h after ECMO, with a decrease in the predicted mortality from 87% to 37% as per the SAPS-II [17,18]. Therefore, ECMO can be a successful resuscitative strategy for near-drowning patients with cardiac or pulmonary failure refractory to conventional ventilator therapy. The encouraging outcomes of this study could be attributed to the early application of ECMO and use of VV ECMO. VV ECMO is safer than VA ECMO in many aspects. Firstly, VV ECMO prevents lower extremity ischemia and complications related to arterial cannulation. Secondly, it prevents upper body hypoxemia, which is fatal to the brain and heart that have already developed preexisting hypoxic damage, especially in patients with good cardiac function [19]. Thirdly, it is less traumatic to blood components and reduces the need for transfusion.

In drowning victims, important predictors for survival are water temperature, submersion time, adequacy of bystander CPR, and EMS response time. However, it is difficult to ascertain these factors. Importantly, the time interval for which the victim is under water is unknown and needs to be estimated. There is no clear cut-off value for the submersion time, although more than 10 min is considered a cut-off point for non-survival [20]. Hypoxic brain damage is the most critical consequence of submersion. A previous study showed that non-reactive pupils and a Glasgow Coma Scale (GCS) score < 5 on arrival at the ICU were the best independent predictors of a poor neurological outcome [21]. The 26-year-old man who fell into a freezing lake after breakage of ice was rescued after an estimated submersion time of 20 min and was transferred to our hospital in a helicopter. He underwent ongoing CPR until ECMO could be started. After being maintained on the ECMO for 160 min and rewarmed to 33°C, he recovered consciousness and achieved ROSC. He was discharged home with intact neurological outcome, despite the submersion time of 20 min, a GCS score of 3, and fixed pupils at the ED. On the contrary, two patients in the present study achieved weaning from ECMO but showed hypoxic brain damage, which was incurred by prolonged hypoxemia in one patient and by longstanding cardiac arrest in the other patient. Irrespective of the initial mental status after drowning, early and aggressive mechanical ventilator therapy and prompt implantation of ECMO, if necessary, are essential to prevent hypoxic brain damage. The 18-year-old man underwent awake ECMO. It was effective in preventing pneumonia and reducing unnecessary sedation when patients were cooperative and a constant bypass blood flow rate was maintained. Continuous renal replacement therapy was implemented in 5 patients and was helpful for preventing fluid overload and aggravation of ARDS [22]. To maintain anticoagulation and reduce bleeding complications, nafamostat mesilate, a synthetic protease inhibitor with a short half-life, was used in 6 patients. Nafamostat mesilate has been used as an anticoagulant for patients undergoing haemodialysis, and its applications are currently expanding to postoperative and serious cases with high risk of bleeding [23]. In this study, there were no complications of bleeding or thrombosis that required an operation or intervention.

Despite our important findings, our study had a few limitations such as a retrospective observational design with a small sample size, the lack of a control group, and no long-term follow-up. Owing to the small number of patients included in this study, we could not perform multivariate statistical analysis. Additionally, we do not have the exact data in relation to drowning such as the time between an accident and call, bystander- initiated CPR, and water temperature, as specified by the Utstein reporting guidelines for uniform reporting of drowning [24].

## Conclusions

The present study demonstrated a 100% rate of weaning from ECMO and discharge rate and a 77.8% survival rate with a favourable neurological outcome at 3 months in patients with ongoing cardiac arrest or ARDS after near-drowning incident. Considering the mean values of severity-scoring system prior to ECMO, such as Murray score of 3.5, SOFA score of 11.4, and SAPS-II of 73, the outcomes of this study were remarkable. ECMO can be used as a resuscitative strategy for near-drowning patients with cardiac or pulmonary failure resistant to conventional ventilator therapy. Its early application is recommended to improve clinical outcomes.

## References

[1] Szpilman D, Joost MD, Bierens LM, Handley AJ, Orlowski JP (2012). Drowning. N Engl J Med.

[2] Nitta M, Kitamura T, Iwami T, Nadkarni VM, Berg RA, Topjian AA, Okamoto Y, Nishiyama C, Nishiuchi T, Hayashi Y, Nishimoto Y, Takasu A (2013). Out-of-hospital cardiac arrest due to drowning among children and adults from the Unstein Osaka Project. Resuscitation.

[3] Ibsen LM, Koch T (2002). Submersion and asphyxia injury. Crit Care Med.

[4] Hasibeder WR (2003). Drowning. Curr Opin Anaesthesiol.

[5] Hill JD, O’Brien TG, Murray JJ, Dontigny L, Bramson ML, Osborn JJ, Gerbode F (1972). Prolonged extracorporeal oxygenation for acute post-traumatic respiratory failure (shock-lung syndrome). Use of the Bramson membrane lung. N Engl J Med.

[6] Bartlett RH, Gazzaniga AB, Jefferies MR, Huxtable RF, Haiduc NJ, Fong SW (1976). Extracorporeal membrane oxygenation (ECMO) cardiopulmonary support in infancy. Trans Am Soc Artif Intern Organs.

[7] Ruttmann E, Weissenbacher A, Ulmer H, Müller L, Höfer D, Kilo J, Rabl W, Schwarz B, Laufer G, Antretter H, Mair P (2007). Prolonged extracorporeal membrane oxygenation-assisted support provides improved survival in hypothermic patients with cardiocirculatory arrest. J Thorac Cardiovasc Surg.

[8] Burford AE, Ryan LM, Stone BJ, Hirshon JM, Klein BL (2005). Drowning and near-drowning in children and adolescents. Pediatr Emerg Care.

[9] Pratt FD, Potgeiter PD (1986). Incidence of secondary drowning after saltwater submersion. Ann Emerg Med.

[10] Bierens JJ, Warner DS (2013). Drowning resuscitation requires another state mind. Resuscitation.

[11] Szpilman D (1997). Near-drowning and drowning classification: a proposal to stratify mortality based on the analysis of 1,831 cases. Chest.

[12] Ballesteros MA, Gutierres-Cuadra M, Munoz P, Minambres E (2009). Prognostic factors and outcome after drowning in an adult population. Acta Anaesthesiol Scand.

[13] Cordell-Smith JA, Roberts N, Peek GJ, Firmin RK (2006). Traumatic lung injury treated by extracorporeal membrane oxygenation (ECMO). Int J Care Injured.

[14] Extracorporeal Life Support Organization: *Adult Respiratory Failure Supplement to the ELSO General Guidelines for Cardiopulmonary Extracorporeal Life Support* [http://elsonet.org/]

[15] Brogan TV, Thiagarajan RR, Rycus PT, Bartlett RH, Bratton SL (2009). Extracorporeal membrane oxygenation in adults with severe respiratory failure: a multi-center database. Intensive Care Med.

[16] Lindskov C, Jensen RH, Sprogoe P, Klaaborg KE, Kirkegaard H, Severinsen K, Lorentsen G, Folkersen L, Ilkjaer S, Pedersen CM (2013). Extracorporeal membrane oxygenation in adult patients with severe acute respiratory failure. Acta Anaesthesiol Scand.

[17] Le Gall J-R, Lemeshow S, Saulnier F (1993). A new simplified acute physiology score (SAPS II) based on a European/North American Multicenter study. JAMA.

[18] Vincent JL, Moreno R, Takala J, Willatts S, De Mendonça A, Bruining H, Reinhart CK, Suter PM, Thijs LG (1996). The SOFA (sepsis-related organ failure assessment) score to describe organ dysfunction/failure. Intensive Care Med.

[19] Sidebotham D, Allen SJ, McGeorge A, Ibbott N, Willcox T (2012). Venovenous extracorporeal membrane oxygenation in adults: Practical aspects of circuits, cannulae, and procedures. J Cardiothorac Vasc Anesth.

[20] Bierens JJ, Knape JT, Gelissen HP (2002). Drowning. Curr Opin Crit Care.

[21] Lavelle JM, Shaw KN (1993). Near drowning: is emergency department cardiopulmonary resuscitation or intensive care unit cerebral resuscitation indicated?. Crit Care Med.

[22] Sutherland SM, Zappitelli M, Alexander SR, Chua AN, Brophy PD, Bunchman TE, Hackbarth R, Somers MJG, Baum M, Symons JM, Flores FX, Benfield M, Askenazi D, Chand D, Fortenberry JD, Mahan JD, McBryde K, Blowey D, Goldstein SL (2010). Fluid overload and mortality in children receiving continuous renal replacement therapy: the prospective pediatric continuous renal replacement therapy registry. Am J Kidney Dis.

[23] Han SJ, Kim HS, Kim KI, Whang SM, Hong KS, Lee WK, Lee SH (2011). Use of nafamostat mesilate as an anticoagulation during extracorporeal membrane oxygenation. J Korean Med Sci.

[24] Idris AH, Berg RA, Bierens J, Bossaert L, Branche CM, Gabrielli A, Graves SA, Handley AJ, Hoelle R, Morley PT, Papa L, Pepe PE, Quan L, Szpilman D, Wigginton JG, Modell JH, Atkins D, Gay M, Kloeck W, Timerman S (2003). Recommended guidelines for uniform reporting of data from drowning: the “Utstein style”. Resuscitation.

